# Influence of Polishing Methods on the Bonding Effectiveness and Durability of Different Resin Cements to Dentin

**DOI:** 10.1155/2018/9189354

**Published:** 2018-02-28

**Authors:** Lingyan Ren, Mingfei Li, Yahui Pan, Xiangfeng Meng

**Affiliations:** Department of Prosthodontics, Nanjing Stomatological Hospital, Nanjing University Medical School, No. 30 Zhongyang Road, Nanjing, Jiangsu 210008, China

## Abstract

The purpose of this study was to investigate the impact of polishing methods on the bonding effectiveness and durability of different resin cements to dentin. The dentin surfaces were either treated with a fine-grit diamond bur (polishing A) or further polished by polishing disks (polishing B), and then they were bonded with any one of the three resin cements, namely, etch-and-rinse, self-etch, and self-adhesive resin cements. After 24-hour or 2-year water storage, a microtensile bond strength (*μ*TBS) test was performed. A scanning electron microscope (SEM) was used to observe the morphology of the smear layer as well as the resin-dentin bonding interface. The results indicated that a thinner smear layer thickness was created by polishing B compared with polishing A. Although self-etch and self-adhesive resin cements achieved a relatively high primary bond strength before water degradation, etch-and-rinse resin cement obtained a stable bond strength during water degradation. The application of an additional polishing procedure could improve the bond strength of self-etch and self-adhesive resin cements.

## 1. Introduction

Resin cements have been wildly used for bonding restorative materials for their excellent mechanical properties, great bond strengths, and realistic esthetics versus conventional cements [1]. Currently, resin cements are classified into three subgroups according to the adhesion protocols, namely, etch-and-rinse, self-etch, and self-adhesive resin cement [2]. Moreover, the treatments of the smear layer are not exactly the same among the three resin cements. There are three steps typically to apply etch-and-rinse resin cement, which are the etching, priming, and bonding. Moreover, the smear layer of this cement is removed by the etching and rinsing steps. As for self-etch resin cement, the smear layer is dissolved and modified by the acid monomers in the primers and further integrated into the hybrid layer and the adhesive layer [3]. In view of the multiple steps and high technical sensitivity of the two types of resin cements, self-adhesive resin cement has attracted lots of interest for its simplified one-step application procedure [4].

Self-adhesive resin cement, designed to bond with the tooth substrate without the need for tooth surface pretreatment and an additional adhesive, has been widely used clinically within the past decade. The composition of the cement includes resin matrix, inorganic fillers, and initiator as well as acid-functionalized monomers which are specifically formulated to endow the cement with self-adhesive properties [4, 5]. The phosphate groups of the acid monomers partially dissolve the smear layer and react with the hydroxyapatite on the superficial dentin [6, 7] with the cement penetrating into the smear layer simultaneously, which becomes incorporated into the polymer network as the cement gets its final curing stage. However, there seems to be an agreement that no hybrid layer or resin tags could be observed between the interface of dentin and cement [5, 8], and no decalcification/infiltration into dentin could be found in several self-adhesive resin cements [8, 9]. On the contrary, for conventional resin cements, that is, etch-and-rinse and self-etch resin cements, the evidence of hybrid layer and resin tags formation is clear [8]. As a result, there are doubts concerning the bond strength and bond durability of self-adhesive resin cement.

Some authors state that the thickness of smear layer has an effect on the bond strength of bonding agents [10, 11], since acid monomers need to penetrate through the smear layer to touch and demineralize the intact dentin, and the insufficient resin impregnation and inadequate resin envelopment of residual smear may accelerate the interface degradation [12]. Actually, the loose and porous thick smear layer that could be a weak part of resin-dentin interface [12] may hamper the bonding strength and durability of resin cements to dentin. However, some researchers state that there is no clear evidence that smear layer thickness has an effect on bond strength [13, 14]. Some in vitro researches utilized various grit sizes of silicon carbide (SiC) abrasive paper for reasons of standardization and ease of preparation to simulate different thickness levels of smear layer [12], while some used carbide bur or diamond bur to create various dentin substrate [15, 16]. So different instruments with similar grit size could create different thickness of smear layer [12]. In our present research, a fine-grit diamond bur and polishing disks were used to grind the dentin surface in order to simulate clinical tooth preparation procedures and therefore to provide a guide for clinical application in selecting a proper and effective dentin surface polishing method for improving the bond strength between dentin and resin cements, especially for the self-adhesive resin cement.

The purpose of our study, therefore, was to investigate whether polishing methods had an effect on the bonding effectiveness and bonding durability of dentin with three resin cements, namely, etch-and-rinse, self-etch, and self-adhesive resin cements. The null hypothesis tested was that the difference in polishing methods and resin cements could not leave an impact on the bonding effectiveness and bonding durability of dentin with resin cements.

## 2. Materials and Methods

### 2.1. Tooth Preparations

Eighty intact, noncarious human third molars were extracted from patients for clinical reasons, and they were collected under the patients' informed consent, according to the local Ethical Committee (The Affiliated Stomatological Hospital, Medical School of Nanjing University, Nanjing, China). The teeth were stored in 0.9% saline solution at 4°C for no longer than 3 months after extraction. The roots of the teeth (1 mm below cementoenamel junction) were embedded in acrylic resin (Unifast Trad, GC, Tokyo, Japan) forming cuboid blocks with the exposure of dental crown to facilitate the fixation of the teeth on the low-speed diamond saw (IsoMet 1000, Buehler, Lake Bluff, IL, USA). The occlusal third of the dental crowns was removed by the low-speed diamond saw under water coolant/lubrication to expose the flat dentin surfaces. Then the teeth were prepared and polished according to the following steps. First, the dentin surfaces were prepared with a regular-grit (105–125 um) high-speed diamond bur (DIATECH, Coltėne/Whaledent AG, Altstätten, Switzerland) under water-cooling. Second, the surfaces were polished by a fine-grit (25 um) high-speed diamond bur (DIATECH, Coltėne/Whaledent AG, Altstätten, Switzerland) under water-cooling (polishing A). Third, half of the dentin surfaces were further polished with slow-speed polishing disks (Sof-Lex, 3M ESPE, Seefeld, USA) from medium-grit to fine-grit to the final superfine-grit (polishing B). All these steps were carried out by one experienced dentist.

Before the bonding procedure, four teeth from each group treated with different polishing methods were randomly selected to evaluate the smear layer's characteristics under a scanning electron microscope (SEM, TM-1000, Hitachi High-Technologies, Hitachinaka, Japan). Each tooth was sectioned transversely into a 2 mm thick slice with a low-speed diamond saw. Afterwards, a transversal groove of 1 mm depth was made with a high-speed diamond bur on the opposite side of the polished dentin surface, and the slice was carefully split into two halves with a bending force applied to the groove. After that, these 16 dentin pieces were processed for SEM including fixation in 2.5% glutaraldehyde for 24 h, dehydration in ascending concentrations (50%, 60%, 70%, 85%, 95%, and 100%) of ethanol for 15 minutes twice, and finally chemical drying with HMDS following the protocol described by Perdigao and others [17]. Then the dentin surfaces of the specimens were sputter-coated with gold and observed under SEM. Half of each tooth was set aside to observe the micromorphology of the dentin surface prepared by separate polishing method and the other half served to measure the thickness of the smear layer through the cross section.

### 2.2. Adhesive Procedures

The remaining 36 teeth of each type of dentin surface were further divided into 3 subgroups of 12 based on 3 types of resin cement: etch-and-rinse (Variolink N, Ivoclar Vivadent, Schaan, Principality of Liechtenstein), self-etch (Multilink N, Ivoclar Vivadent, Schaan, Principality of Liechtenstein), and self-adhesive (Multilink Speed, Ivoclar Vivadent, Schaan, Principality of Liechtenstein). Their compositions, shades, manufacturers, and batch numbers were described in Table 1. For each type of resin cement, a specific bonding procedure was applied according to the respective manufacturers' instructions.

For etch-and-rinse resin cement, the dentin surfaces were cleaned and etched with 37% phosphoric acid gel (Total Etch, Ivoclar Vivadent, Schaan, Principality of Liechtenstein) for 10–15 s, rinsed for 10 s with water, and softly dried with oil- and water-free air leaving the dentin surfaces visibly moist. Syntac primer was applied on the dentin for 15 s and dispersed with an air syringe. Then Syntac adhesive was applied for 10 s and thoroughly dried with blown air. Afterwards, Heliobond was applied and blown to a thin layer. Variolink N Base and Catalyst were mixed in a 1 : 1 ratio for 10 s and immediately applied on the dentin surfaces.

For self-etch resin cement, the dentin surfaces were cleaned with water and dried with oil- and water-free air leaving the dentin surfaces visibly moist. Multilink N Primers A and B were mixed in a 1 : 1 ratio and applied on dentin surfaces for 15 s. The mixed primer was dried with blown air. Then Multilink N was dispersed from the automix syringe and applied onto the dentin.

For self-adhesive resin cement, the dentin surfaces were cleaned with water and dried with oil- and water-free air leaving the dentin surfaces visibly moist. Multilink Speed was ejected from the automix syringe and applied onto the dentin.

Seventy-two resin composite cylinders (Filtek Z250, 3M ESPE, Seefeld, USA) with a thickness of 4 mm and a diameter of 7 mm were formed and irradiated by a LED light unit with the intensity of 800 mW/cm^2^ (Bluephase C8, Ivoclar Vivadent, Schaan, Principality of Liechtenstein) for 20 s per side. Then the resin composite cylinders were immediately bonded on the dentin surfaces under a fixed pressure of 5 N simulating the finger pressure clinically. Each resin-dentin bonding specimen was light cured with a light intensity of 800 mW/cm^2^ for 20 s per direction. Afterwards, the bonding specimens were stored in distilled water at 37°C for 24 hours.

After the bonding procedure, four teeth from each group treated with different types of resin cement were used to evaluate the resin-dentin interfaces under SEM. Every tooth was longitudinally sectioned into two halves with a low-speed diamond saw to expose the bonding interface. Then the specimens were etched with 10% phosphoric acid for 3 s and rinsed with deionized water for 15 s followed by immersion in 5.25% sodium hypochlorite solution for 10 minutes. The specimens were rinsed and kept in a desiccator for 24 hours and then were sputter-coated with gold and observed under SEM.

### 2.3. Microtensile Bond Strength Testing

**Figure 1:**
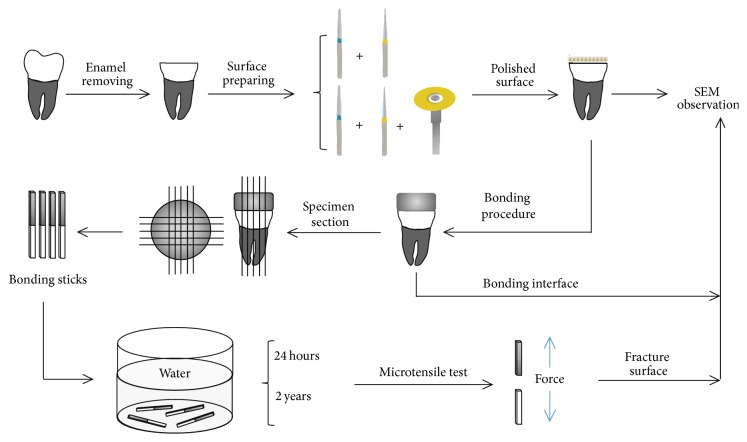
Schematic illustrations showing the experimental design of the study.

After storage, 8 of the 12 teeth in each resin cement group were longitudinally sectioned in both “*x*” and “*y*” directions across the bonded interface with a low-speed diamond saw under water-cooling to obtain resin-dentin bonding sticks with a cross-sectional area of approximately 0.9 mm^2^. About 8–10 sticks from the central area of each tooth were obtained for microtensile tests to try to minimize substrate regional variability. Forty sticks whose dentin part was longer than 4 mm were selected from these sticks for each group and were further randomly divided into 2 subgroups (*n* = 20) according to 2 different storage times, respectively, 24 hours and 2 years, in distilled water (Figure 1).

The bonding specimens were fixed to a microtensile bond strength testing device (T-61010K, BISCO Inc., Schaumburg, IL, USA) with cyanoacrylate glue. Tensile forces were implemented at a crosshead speed of 0.5 mm/min until failure occurred. After sticks fracture, the dimensions of the bonding area were measured by a vernier caliper and were used to calculate the *μ*TBS (in MPa) by dividing the imposed force (in N) at the time of fracture by the bonded area (in mm^2^).

**Figure 2:**
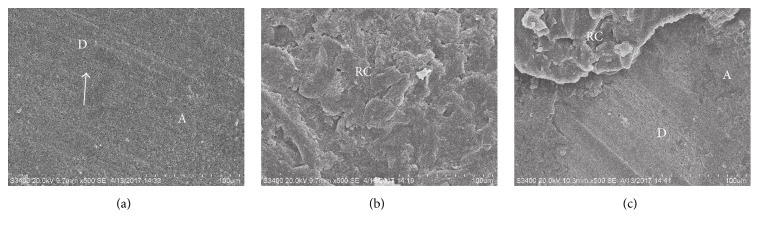
Representative SEM micrographs of the dentin side of the fractured specimen. (a) Adhesive failure, failure within dentin/resin cements interface; (b) cohesive failure, failure solely within resin cements; (c) mixed failure, partially cohesive failure within resin cements with some adhesive failure. A: adhesives; D: dentin; RC: resin cements. The dentinal tubules on the dentin surface (white arrow).

To determine the failure modes, both the dentin and composite halves of the fractured specimens were observed with an integrated microscope (30x magnification, SMZ1500, Nikon Corp., Tokyo, Japan). The failure modes were divided into three types shown in Figure 2: (a) adhesive failure (failure within dentin/resin cements interface); (b) cohesive failure (failure solely within resin cements); (c) mixed failure (partially cohesive failure within resin cements with some adhesive failure).

After the microtensile test, some of the fractured specimens were rinsed, dried, dehydrated, and sputter-coated as mentioned above and observed under SEM.

### 2.4. Statistical Analysis

Statistical analysis was performed with SPSS 20.0 software. Means and standard deviations of microtensile bond strengths measured in Megapascals (MPa) were calculated for each group. Within each storage time, a factorial design was performed to analyze the differences in bond strength between resin cements versus polishing methods and the interactions between the two factors. Because of the significant interaction between resin cements and polishing methods irrespective of the storage time, it is necessary to analyze the simple effect of the two factors. A one-way ANOVA and the Turkey test were used to analyze the difference of bond strength between resin cements while an independent sample *t*-test was utilized to test the difference of bond strengths between groups according to water storage time or polishing methods. The significance level was set at 0.05 for all statistical tests.

## 3. Results

### 3.1. The Observation of Smear Layer Characteristics of Dentin

**Figure 3:**
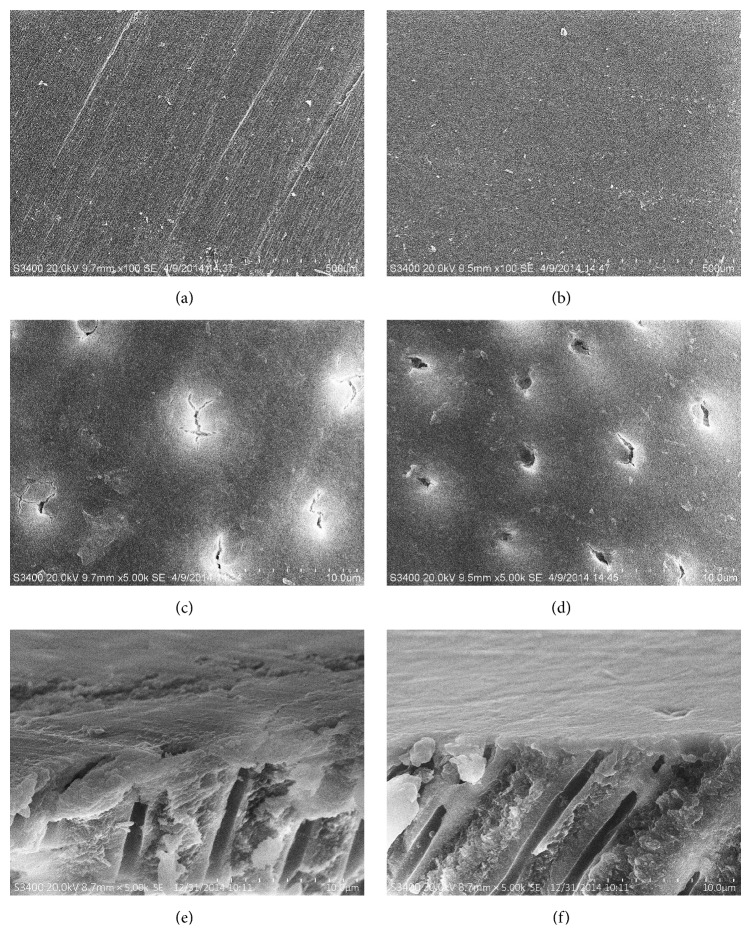
Representative SEM photographs of the smear layer created by two polishing methods. The morphology of smear layer ground by polishing A (a) and polishing B (b) at 100 times magnification. The dentinal tubule openness created by polishing A (c) and polishing B (d) at 5,000 times magnification. The thickness of smear layer produced by polishing A (e) and polishing B (f) at 5,000 times magnification.

The smear layer created by two polishing methods appears in Figure 3. Many regularly distributed scratches left by the diamond bur with the entire surface covered with a deposit of debris could be observed on the dentin surface ground by polishing A (Figure 3(a)). For groups ground with polishing discs, the surface was smoother and flatter without visible scratches, and the evenly and sparsely distributed debris was observed (Figure 3(b)).

The dentin surface was further magnified to observe the dentinal tubules morphology. The openness of the tubules was larger in polishing B (Figure 3(d)) than in polishing A (Figure 3(c)).

The thickness of the smear layer created by two polishing methods could be evaluated under SEM from the cross section of the dentin. Polishing A produced a thicker smear layer with longer smear plugs clogging in the dentinal tubules (Figure 3(e)) than polishing B. The dentin surface of polishing B was flatter and more homogeneous (Figure 3(f)) than polishing A.

### 3.2. The Observation of Resin-Dentin Bonding Interface

**Figure 4:**
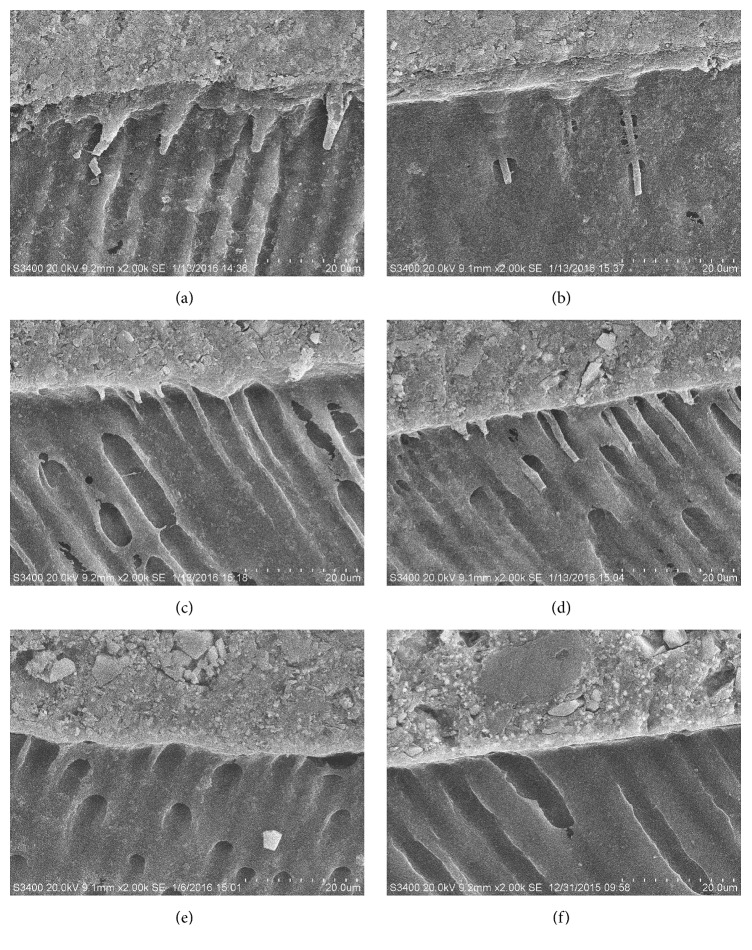
Representative SEM photographs of the resin-dentin interface treated with three resin cements at 2,000 times magnification. Etch-and-rinse resin cement with polishing A (a) and polishing B (b). Self-etch resin cement with polishing A (c) and polishing B (d). Self-adhesive resin cement with polishing A (e) and polishing B (f).

In both etch-and-rinse and self-etch resin cements, the penetration of the adhesive into the dentinal tubules was clearly observed (Figures 4(a)–4(d)), while no visible resin tags were observed in self-adhesive resin cement (Figures 4(e) and 4(f)). The etch-and-rinse resin cement formed consistent resin tags in both polishing A and B groups (Figures 4(a) and 4(b)). For self-etch resin cement, denser and longer resin tags were discerned in polishing B group (Figure 4(d)), while shorter resin tags with some voids along the bonding interface were observed in polishing A group (Figure 4(c)).

### 3.3. Microtensile Bond Strength

The means of *μ*TBS and standard deviations (SD) for each test group were summarized in Table 2.

The results of the factorial design indicated that, within either water storage time, types of resin cements and polishing methods had significant effects on *μ*TBS (*p* < 0.01), and the interactions between the two factors were significant (*p* < 0.01).

After 24-hour water storage, the *μ*TBS of self-etch resin cement was significantly higher than that of etch-and-rinse and self-adhesive resin cement in both polishing A and B groups (*p* < 0.01). The *μ*TBS of etch-and-rinse resin cement had no significant difference between polishing A and polishing B, while in both self-etch and self-adhesive resin cements (*p* > 0.05) the *μ*TBS produced by polishing B was significantly higher than that created by polishing A (*p* < 0.01).

After 2-year water storage, the *μ*TBS of etch-and-rinse resin cement had no significant change compared with that before water degradation in both polishing methods (*p* > 0.05), while the *μ*TBS of self-etch and self-adhesive resin cements showed significant decrease in both polishing methods (*p* < 0.05). Besides, the *μ*TBS of self-adhesive resin cement had no significant difference between polishing A and polishing B (*p* > 0.05), while self-etch resin cement had higher *μ*TBS in polishing B than polishing A (*p* < 0.01).

### 3.4. The Observation of Bond Failure Modes

The failure mode counts of all test groups are visible in Table 3. Most of the specimens in each group were with adhesive failure that occurred at the interface of dentin and resin cement no matter how long the water storage time was.

## 4. Discussion

The results of the fracture mode counts in this study showed that most of the fracture occurred at the interface between dentin and resin cements, which meant that the *μ*TBS values were close to the true resin-dentin bond strength. So, according to our results, the null hypothesis that polishing methods and types of resin cements had no impact on the bonding effectiveness and bonding durability proved to be false.

In this study, etch-and-rinse resin cement obtained an initial bond strength of around 25 MPa. The bonding force of etch-and-rinse cement mainly relies on micromechanical retention as the phosphoric acid can sufficiently dissolve the smear layer and demineralize dentin, with the resin monomers infiltrating into the collagen fibrils and dentinal tubules to form the hybrid layer and the resin tags, respectively (Figures 4(a) and 4(b)), which provides a micromechanical retention between resin and dentin [18, 19]. Many studies stated that etch-and-rinse bonding system could obtain a higher initial bond strength to dentin with proper bonding protocols [14, 20]. The reasons for the different results in the current study and cited studies may be the differences in the preparation of dentin surface [21], the bonding protocols [6] and materials [22], the curing mode [23], and so on.

Self-etch resin cement obtained an initial bond strength of around 60–70 MPa. In self-etch resin cement, Multilink N Primer used in this study for conditioning the dentin surface prior to Multilink N was a one-step self-etch bonding agent. The phosphonic acid monomer content in the Multilink N Primer B is up to 48.1% according to the Scientific Documentation of Multilink N. Therefore, the acidic monomers can dissolve the smear layer and the underlying sound dentin to form a micromechanical retention as shown in the SEM image (Figures 4(c) and 4(d)). Moreover, some studies have clarified that the monomers in self-etch bonding agent could form chemical bonds with the hydroxyapatite on dentin surface [24, 25].

The above hypothesis could explain the great 24-hour bond strength of self-adhesive resin cement as well. The results of this study suggested that the initial bond strength of self-adhesive resin cement was as high as that of etch-and-rinse system or even higher when polishing B was applied with a value of about 35 MPa. The phosphoric acid monomer content in Multilink Speed Catalyst was only 3.1% (Scientific Documentation of Multilink Speed), which was significantly lower than that in Multilink N Primer. Thus the content of acidic monomers was too low to dissolve the smear layer effectively and form a hybrid layer [4, 26]. Although self-adhesive resin cement obtained insufficient micromechanical retention, some studies demonstrated that additional chemical interaction between the phosphoric acid monomer and hydroxyapatite accounts for the bond strength of self-adhesive resin cement according to the adhesion-decalcification concept [27, 28]. This suggests that chemical bonding could be a plausible explanation for the good initial bond strength of self-etch and self-adhesive systems.

It is worth noting that etch-and-rinse resin cements produced constant bond strength during water storage while self-etch and self-adhesive resin cement suffered a sharp decrease. In some in vitro and in vivo studies, the bonding interfaces of self-etch and self-adhesive system were thought to act as permeable membranes [29–31], permitting water through the adhesive layer. This may be due to the fact that one-step self-etch and self-adhesive resin cements are mixtures of hydrophilic and hydrophobic components which results in a high hydrophilicity of the adhesive layer that absorbs water from oral environment and dentinal tubules [28]. Generally, the increase in water sorption may contribute to accelerated degradation of resin-dentin bonds [28, 32]. Additionally, the hydrophobic bonding agent such as Heliobond in Variolink N may play an important role in the long-term bonding performance because a hydrophobic adhesive layer could seal the dentin well [18, 28, 31]. Conversely, one-step self-etch system lacked a hydrophobic resin layer on the conditioned dentin surface. Furthermore, the hydrolysis of the chemical bonds may be a plausible explanation for the poor resistance to water degradation of self-etch and self-adhesive resin cements. Especially for self-adhesive resin cement, the bond strength decreased to a value lower than 10 MPa after 2 years of water storage in both polishing groups. The results forced us to doubt that chemical bond on which self-adhesive depended was more susceptible to hydrolysis. The instability of calcium phosphate/carboxylate compounds may lead to the degradation and debonding of the resin-dentin bonds [28]. Although some scholars suggest that the chemical bond is important to promote bonding durability [24, 33], some of the studies, however, suggest different acidic monomers can form more or less stable chemical bonds [34, 35]. Usually, bonding systems and cements containing 10-MDP are supposed to promote more stable chemical bonding [35, 36]. Actually, the structure [37, 38], the purity and content of acidic monomers [39], and the formulation of the product are various among the products made by different manufactures, which would affect the bonding effectiveness and durability. At present, it has not been clarified yet whether the chemical bonds can improve the bonding durability or not. Because of the limitations of our study, our observation that mechanical retention seems more stable than chemical bonds during water degradation requires verification while conducting further experiments.

Our study and some other studies showed that the morphology of smear layer created by different polishing methods had no significant effect on the bond strength of etch-and-rinse resin cement [13, 40]. This may be due to the fact that the smear layer and smear plugs were dissolved by phosphoric acid and then washed away after rinsing procedure. However, the morphology of smear layer produced by two polishing methods had a significant effect on the bond effectiveness of the self-etch and self-adhesive resin cements.

Additional polishing procedures improved the initial 24-hour bond strength of self-etch and self-adhesive resin cements, especially the latter one. Some previous studies demonstrated that for self-etch and self-adhesive resin cements the acidic monomers were rapidly neutralized while diffusing through the thick smear layer [11], and fewer acidic monomers could reach and demineralize the intact dentin surface underneath the smear layer [3]. As seen in the SEM image (Figure 4(d)), the resin tags were denser and longer than that in Figure 4(c), since a thinner smear layer was easier for acidic monomers to penetrate through. Some cited studies stated that applied preparation methods on dentin using such as SiC paper [12] or carbide bur [15] and the denseness of smear layer [3] can also affect the bond strength. Considering the limitations of this study, the effect of these factors on bond strength needs to be verified in further studies.

## 5. Conclusion

In conclusion, the resin-dentin interface treated by etch-and-rinse resin cement was the most stable during water degradation. Furthermore, polishing methods could improve the bonding effectiveness of dentin with self-etch and self-adhesive resin cements.

## Figures and Tables

**Table 1:** Materials used in this experiment.

Product name (manufacturer)	Material	Main composition^a^	Batch number
Variolink N (Ivoclar Vivadent, Schaan, Principality of Liechtenstein)	Syntac primer	Triethylene glycol methacrylate, PEGDMA, maleic acid, and ketone in an aqueous solution	R96210
	Syntac adhesive	PEGDMA and glutaraldehyde in an aqueous solution	R79315
	Heliobond	Bis-GMA, TEGDMA, stabilizers, and initiators	V03480

Variolink N (Ivoclar Vivadent, Schaan, Principality of Liechtenstein)	Resin cement	Monomer matrix: Bis-GMA, UDMA, TEGDMA Fillers: 46.7% wt, 0.7 *μ*m mean particle size, barium glass, ytterbium trifluoride, Ba-Al-F-Si glass, spheroid mixed oxide Additional contents: initiators, stabilizers, pigments	T32910 yellow(universal), A3

Multilink N (Ivoclar Vivadent, Schaan, Principality of Liechtenstein)	Primer	Primer A is an aqueous solution of initiators Primer B contains HEMA, phosphonic acid acrylate, methacrylate monomers, and stabilizers	S34663

Multilink N (Ivoclar Vivadent, Schaan, Principality of Liechtenstein)	Resin cement	Monomer matrix: dimethacrylate and HEMA Fillers: 0.9 *μ*m mean particle size, 40 vol%, barium glass, ytterbium trifluoride, and spheroid mixed oxide	T18945 transparent

Multilink Speed (Ivoclar Vivadent, Schaan, Principality of Liechtenstein)	Resin cement	Monomer matrix: dimethacrylates and phosphoric acid monomers Fillers: mean particle size 5 *μ*m, 40 vol%, barium glass, ytterbium trifluoride, copolymer, and silicon dioxide Additional contents: catalysts, stabilizers, and pigments	S05050 translucent

^a^Composition as provided by the manufacturers: TEGDMA, triethylene glycol dimethacrylate; PEGDMA, polyethylene glycol dimethacrylate; Bis-GMA, bisphenol A glycidyl methacrylate; HEMA, hydroxyethyl methacrylate; UDMA, urethane dimethacrylate.

**Table 2:** Microtensile bond strengths (mean ± SD, MPa) for all specimen groups.

Resin cement	Polishing method	Water storage time	
		24 hours	2 years
Etch-and-rinse	A	24.4 ± 7.6^aA^	25.6 ± 7.8^aA^
	B	24.4 ± 9.0^aA^	23.5 ± 7.3^aA^
Self-etch	A	60.8 ± 14.4^bA^	15.7 ± 8.5^bB^
	B	71.3 ± 11.8^cA^	28.0 ± 12.6^aB^
Self-adhesive	A	21.9 ± 5.8^aA^	7.3 ± 5.0^cB^
	B	36.5 ± 6.3^dA^	8.8 ± 2.6^cB^

Means with same letters are not significantly different by Turkey's test (*p* > 0.05). Means with the same lowercase letters within same column are not statistically different, and means with the same uppercase letters within same row are not statistically different.

**Table 3:** Failure mode counts of all the specimens after microtensile test.

Resin cement	Polishing method	24-hour water storage			2-year water storage		
		(a)	(b)	(c)	(a)	(b)	(c)
Etch-and-rinse	A	17	0	3	15	3	2
	B	19	0	1	16	2	2
Self-etch	A	18	1	1	18	1	1
	B	18	0	2	17	2	1
Self-adhesive	A	20	0	0	19	0	1
	B	18	0	2	18	0	2

Failure modes: (a) adhesive failure (failure within dentin/resin cements interface); (b) cohesive failure (failure solely within resin cements); (c) mixed failure (partially cohesive failure within resin cements with some adhesive failure).
